# Complete genome sequence and description of *Escherichia coli* isolated from fresh vegetables in Baghdad, Iraq

**DOI:** 10.1016/j.jgeb.2026.100699

**Published:** 2026-05-02

**Authors:** Zaid Akram Thabit, Yaseen Ismael Mamoori, May Ridha Jaffer, Ibarahim Abdulla Ahmed, Zahraa Abdulkareem AlShaheeb, Athraa Abdulhadi, Safaa A.S. Al-Qaysi, Sana M.H. AL-Shimmary

**Affiliations:** a Biotechnology Research Center, Environmental Biotechnology Department, Al-Nahrain University, Jadriya, Baghdad, Iraq; b Department of Molecular and Medical Biotechnology, College of Biotechnology, Al-Nahrain University, Jadriya, Baghdad, Iraq; c Department of Forensic Biology, Higher Institute of Forensic Sciences, Al-Nahrain University, Jadriya, Baghdad, Iraq; d Department of Applied Biology, College of Biotechnology, Al-Nahrain University, Jadriya, Baghdad, Iraq; e Department of Biology, College of Science for Women/University of Baghdad, Baghdad, Iraq

**Keywords:** *Escherichia coli*, Whole-genome sequencing, Vegetables, Antimicrobial resistance genes, Next-generation sequencing

## Abstract

Raw vegetables and fresh vegetables, canned tomato salads, and cooked sauces are common and good ways to help make up a healthy plate of food. However, the number of foodborne disease outbreaks associated with fresh fruits and vegetables has increased, with *E. coli* being the most commonly identified pathogen. Some forms of *E. coli* are pathogens that can cause serious health problems in humans, such as hemolytic uremic syndrome, hemorrhagic colitis, and diarrhea. The complete genome sequence of *E. coli* isolated from vegetables in Baghdad, Iraq, is described. Fifty vegetable samples (collected from various markets) were subjected to tests for *E. coli* contamination in the present investigation. Bacterial isolates were characterized by their morphology, culture, and molecular identity, including whole-genome sequencing (next-generation sequencing). The findings indicated that *E. coli* was present in 3 of 50 samples (2 cucumbers and 1 celery), corresponding to 6% of all samples. On the basis of multilocus sequence typing, the three isolates belonged to two sequence types, ST223 and ST1737. Genome assembly indicated that the *E. coli* isolates had a genome size of ∼4.8 Mb and a GC content of ∼50.7%. WGS also revealed that the bacterial isolates exhibited various antibiotic resistance mechanisms, including efflux pumps, enzymatic modification, and regulatory determinants. Phylogenetic tree analysis revealed that these sequenced isolates are closely related to *E. coli* O104:H4. The results of this study demonstrate the need for ongoing genomic research to track the emergence of bacterial isolates and their potential impact on public health.

## 1 Introduction

*Escherichia coli is a* bacterium found in diverse environments, such as the gut of humans and animals.^1^ Some *E. coli* isolates are beneficial, whereas others, such as enterotoxigenic, enteropathogenic, and Shiga toxin-producing *E. coli*, are pathogenic and can cause severe foodborne diseases,^2^, ^3^ The flexibility and adaptability of *E. coli* isolates enable them to proliferate and survive under various environmental conditions, including adhering to plant surfaces, thereby rendering traditional sanitation protocols ineffective,^4^.

The contamination of fresh produce, including cucumbers, cabbage, lettuce, and celery, with pathogenic *E. coli* is an important food safety concern. Vegetables can transmit pathogens when they are irrigated with contaminated water,^4^, ^5^ Furthermore, poor hygiene during transportation and storage, as well as insufficient washing, doubles the risk of contamination.^3^

Whole-genome sequencing (WGS) using Sanger and next-generation sequencing (NGS) was used to study and characterize pathogens, including viruses (Manoori et al., 2024) and bacteria.^6^ Many recent studies have demonstrated the importance of whole-genome sequencing for the surveillance and identification of foodborne *E. coli* isolates. Genome analysis has revealed high genetic diversity and numerous clinically important determinants of virulence and antimicrobial resistance,^7^, ^8^ Additionally, WGS is now considered an important tool for transmission tracking, outbreak detection, and risk assessment in food safety,^9^, ^10^ Many studies have used WGS to identify biofilm-associated genes, stress responses, and persistence in plant environments,^3^, ^11^ Therefore, there is a strong reliance on genomic surveillance to monitor foodborne pathogens and support the expansion and application of evidence-based public health measures.

The detection and surveillance of *E. coli* associated with fresh produce pose many challenges despite the development of molecular diagnostic tools. Traditional methods may lack precision in detecting antimicrobial resistance and virulence genes, and genomic surveillance of foodborne pathogens remains limited in many countries, particularly in Iraq. Genomic data on the diversity and resistance profiles of *E. coli* isolated from vegetables in Iraq are scarce, limiting effective monitoring and risk assessment. Therefore, the objective of the current study was to perform WGS on *E. coli* isolates recovered from vegetables in Iraq to provide baseline data for food safety and antimicrobial resistance surveillance.

## 2 Materials and methods

### 2.1 Sample collection

*Escherichia coli* isolates were recovered from 50 fresh vegetable samples (10 each of tomato, cucumber, cabbage, lettuce, and celery). The collection period was from January 2022 to February 2022. The samples were collected from several markets in Baghdad, Iraq, in sterile bags and transferred to the laboratory, where they were handled upon arrival.

### 2.2 Bacterial isolation

Bacterial isolates were recovered from vegetable samples by weighing 10 g of each sample and transferring them to a sterile flask containing 90 mL of sterile distilled water. A blender was used to homogenize the samples, and the samples were centrifuged at 3000*g* for 5 min. Serial dilutions (10^−1^ to 10^−5^) from each homogenized sample were prepared, and 100 µL of each sample was spread onto Hichrome agar as well as Eosin methylene blue media (Himedia, India). The pales were incubated at 37°C for 24 h, and the *E. coli* isolates were initially identified and selected on the basis of the colony color on both media.

### 2.3 Antibiotic sensitivity testing

*Escherichia coli* isolates were assessed for antimicrobial susceptibility (AST) using the VITEK2 system (bioMérieux, France) according to the recommended instructions. Several bacterial colonies cultivated on Mueller‒Hinton agar for 24 h were selected and suspended in 0.45% NaCl solution to obtain a turbidity equivalent to 0.5 McFarland standard, which was measured with a densitometer. The standardized bacterial suspension was loaded into VITEK 2 AST-GN cards specific for gram-negative organisms. Interpretation of AST results, including classification as susceptible (S), intermediate (I), or resistant (R), was performed in accordance with the Clinical and Laboratory Standards Institute (CLSI) guidelines. The antimicrobial agents tested included representatives of major antibiotic classes commonly used to treat *E. coli* infections, such as β-lactams, aminoglycosides, fluoroquinolones, carbapenems, and sulfonamides. The resulting MIC values were recorded and used to determine the isolates’ phenotypic resistance profiles and to assess antimicrobial resistance genes identified through whole-genome sequencing.

### 2.4 Molecular identification of bacterial isolates

Genomic DNA from three bacterial isolates was extracted using the Wizard Genomic DNA Purification Kit (Promega, USA) according to the manufacturer’s instructions. DNA concentration and purity were estimated by using a NanoDrop 2000c spectrophotometer (Thermo Fisher Scientific, USA). Three bacterial isolates were identified using species-specific *uidA* gene primers (F: CGCCGATGCAGATATTCGTA, R: CTGCCAGTTCAGTTCRTTGT) as described by Brons et al.^12^ Amplification was performed using PCR in a total reaction volume (20 µl) comprising 10 µl of GoTaq G2 Green Master Mix (2X, Promega, USA), 0.5 µl of each primer (10 µM, Macrogen, Korea), and 2 µl of template DNA. The reaction mixture was subjected to thermal cycling using a TC-Plus thermal cycler (Techne, UK). The cycling conditions included initial denaturation at 95°C for 2 min, followed by 35 cycles of 95°C for 30 s, 58°C for 30 s, and 72°C for 30 s, and a final step at 72°C for 5 min. The amplified products were resolved on a 1% agarose gel, electrophoresed in 1x TBE buffer at 75 V for 70 min, visualized, and photographed using a documentation system (Bio-Rad, USA).

### 2.5 Detection of virulence genes

PCR was performed to detect the virulence genes associated with pathogenic E. coli, including stx1, stx2, and eae. The following primer sets were designed in this study: *sxt1* (F: GAATTGCCCCCAGAGTGGAT, R: ATGTGTCCGGCAGATGGAAG), *sxt2* (F: ATATCAGTGCCCGGTGTGAC, R: AAACGCAGAACTGCTCTGGA), and *eae* (F: AGTCGCTTTAACCTCAGCCC, R: TTCAGCATAGCGGAAGCCAA). PCR amplification was carried out in a total reaction volume of 20 µL, containing 10 µL of 2 × PCR master mix, 0.5 µL of each primer (10 µM), 2 µL of template DNA, and 7.5 µL of nuclease-free water. Amplification was performed in a thermal cycler under the following conditions: 95°C for 2 min, followed by 35 cycles of 95°C for 30 s, 60°C for 30 s, and 72°C for 30 s, with a final step at 72°C for 5 min. The PCR products were analyzed by electrophoresis on a 1% agarose gel stained with ethidium bromide and visualized and photographed using a documentation system.

### 2.6 DNA extraction and whole-genome sequencing

Genomic DNA from three *E. coli* isolates was extracted using the ABIOpure Total DNA Kit (AllianceBio, USA) according to the manufacturer’s instructions. The concentration and purity of the extracted DNA were assessed using a Quantus fluorometer (Promega, USA). Whole-genome sequencing (WGS) libraries were prepared according to the reference guide of the Illumina DNA Prep kit (Document #1000000025416 v10, Illumina, USA). WGS for three bacterial isolates was performed on a MiSeq sequencer using the MiSeq Reagent V2 Kit (300-cycle, Illumina, USA), generating paired-end reads. The resulting FASTQ sequence files were analyzed using different computational tools.

### 2.7 Genome assembly

Genome assembly of the three E. coli isolates was performed using the SPAdes assembler tool.^13^ The assembled genomes were uploaded to the web-based Bacterial and Viral Bioinformatics Resource Center (BV-BRC).^14^ Gene and coding sequence annotations were performed using the RAST tool kit (RASTtk).^15^ The annotations included hypothetical proteins and functional assignments, which were linked to Gene Ontology (GO) terms,^16^ mapped to KEGG pathways,^17^ and assigned Enzyme Commission (EC) numbers.^18^ PATRIC annotation revealed two types of protein families: cross-genus protein families (PGFams) and genus-specific protein families (PLFams).^19^ A circular graphical representation of the genome annotations was generated. From inner to outer rings: GC skew, GC content, CDS with homology to identified virulence factors, CDS with homology to recognized antimicrobial resistance genes, RNA genes, reverse-strand CDS, forward-strand CDS, and contigs. The CDS colors on the forward and reverse strands corresponded to the subsystems to which these genes belong. A subsystem is a group of proteins that collectively perform an exact biological function or form a structural complex.^20^ The BV-BRC annotation also involved a unique analysis of subsystems in each genome.

### 2.8 Genes with specialty

Annotation of numerous genes revealed a homology to identified transporters,^21^ virulence factors,^22^, ^23^ drug targets,^24^, ^25^ and antibiotic resistance genes.^26^

### 2.9 Antimicrobial resistance genes and MLST

The Genome Annotation Service at BV-BRC uses a k-*mer*-based method to identify antimicrobial resistance (AMR) genes and relies on the curated database of representative AMR gene sequence variants from BV-BRC. Each identified AMR gene is annotated with its functional role, the general mechanism of antibiotic resistance, the associated drug class, and, in some cases, the specific antibiotic it provides resistance against. Multilocus sequence typing (MLST) of the three *E. coli* isolates was performed using the Staramr pipeline to determine their sequence types (STs).^27^

### 2.10 Phylogenetic analysis

Phylogenetic analysis of bacterial isolates was performed using representative genomes and their closest references, as determined with Mash/MinHash.^28^ PATRIC global protein families (PGFams) from these genomes were selected to determine the phylogenetic placement of the analyzed genome. Alignment using MUSCLE was performed on protein sequences from these families,^29^ and the corresponding nucleotide sequences were mapped to the protein alignment. The resulting amino acid and nucleotide alignments were concatenated into a single data matrix and analyzed using RAxML.^30^ Fast bootstrapping^31^ was employed to generate support values for the phylogenetic tree.

### 2.11 Data availability and number of accessions

The whole-genome sequencing data generated in this study have been deposited in the National Center for Biotechnology Information (NCBI) and GenBank repositories and are publicly available. The accession numbers for the sequenced E. coli isolates are as follows: Eco 5: GenBank accession no. JBNMCQ000000000; Eco 6: GenBank accession no. JBNMCP000000000; and Eco 8: GenBank accession no. JBNMCO000000000.

## 3 Results

### 3.1 Bacterial isolates and antibiotic sensitivity testing

Analysis of 50 vegetable samples revealed three confirmed *E. coli* isolates (6%) on the basis of their morphological features, the VITEK 2 system, and other molecular tools. These isolates were selected for further antimicrobial susceptibility testing and whole-genome sequencing analysis.

The AST results for the three *E. coli* isolates revealed complete susceptibility to all tested antimicrobial agents, with no resistance detected in the testing panel.

### 3.2 Molecular detection of bacterial isolates

**Fig. 1:**
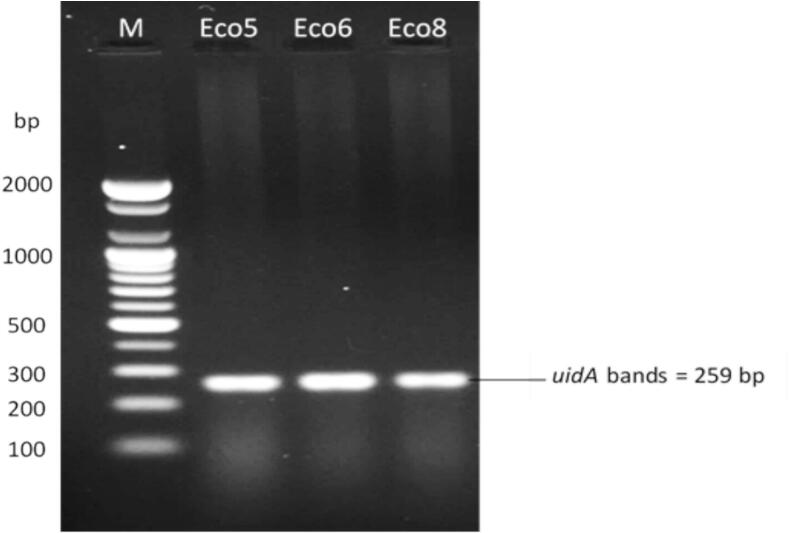
Gel electrophoresis of three amplicons for the uidA gene in three E. coli isolates (Eco5, Eco6, and Eco8). Electrophoresis was performed on a 1.2% agarose gel at 75 V for 70 min.

The primer set for the *uidA* gene was used to detect the three *E. coli* isolates and produced amplicons of the expected size (259 bp) with no nonspecific reactions, as shown in Fig. 1.

### 3.3 Detection of virulence genes using PCR

PCR screening was used to detect the major virulence genes of pathogenic E. coli, including *stx1*, *stx2*, and *eae*. No amplification products were detected for any of the tested virulence genes in the three E. coli isolates.

### 3.4 Multilocus sequence typing (MLST)

It is a robust method to characterize bacterial strains on the basis of variations in housekeeping genes. Analysis of the three Escherichia coli isolates (Eco5, Eco6, and Eco8) revealed significant genetic diversity, with the isolate Eco5 belonging to sequence type (ST) 223 and the isolates Eco6 and Eco8 grouped in ST1737 (Table 1).

**Table 1:** MLST of *E. coli* isolates.

*E. coli* isolate	Sequence Type	Locus 1	Locus 2	Locus 3	Locus 4	Locus 5	Locus 6	Locus 7
Eco5	223	adk(6)	fumC(4)	gyrB(4)	icd(18)	mdh(24)	purA(8)	recA(14)
Eco6 and Eco8	1737	adk(6)	fumC(23)	gyrB(32)	icd(88)	mdh(9)	purA(8)	recA(7)

### 3.5 Genome assembly and annotation

The genome assembly results for the three *E. coli* isolates revealed high-quality assemblies for Eco6 and Eco8, with slight fragmentation in Eco5. *E. coli* isolates have genome lengths of ∼4.8 Mb, which are within the expected range for *E. coli* (∼4.5–5.5 Mb). The GC content of the tested bacterial isolates was ∼50.7%, confirming the accuracy of the assemblies, as shown in Table 2.

**Table 2:** Genome assembly data of three *E. coli* isolates.

Feature	Eco5	Eco6	Eco8
Contigs	206	123	147
GC Content %	50.71	50.74	50.68
Plasmids	None	None	None
Contig L50	18	10	11
Genome length (bp)	4,864,282	4,771,867	4,801,964
Contig N50	93,843	154,422	154,423
Chromosome	1	1	1

The genome annotation results for the three *E. coli* isolates revealed differences in CDSs, tRNAs, repeat regions, and rRNA gene counts (Table 3).

**Table 3:** Genome annotation of the three E. coli isolates.

Feature	Eco 5	Eco6	Eco8
CDS	4,893	4,733	4,756
tRNA	53	80	65
Repeat Regions	32	17	17
rRNA	2	6	5

Among the CDSs in the genomes, Eco5 had the highest CDS count (4893), while Eco6 had the lowest (4733). Eco6 had a significantly greater tRNA gene count (80) than did Eco5 (53) and Eco8 (65). Eco5 had the greatest number of repeat regions (32), whereas Eco6 and Eco8 had fewer (17 each), suggesting relatively stable genomes. The rRNA gene count is highest in Eco6 (6), followed by Eco8 (5) and Eco5 (2).

### 3.6 Functional genomics

**Fig. 2:**
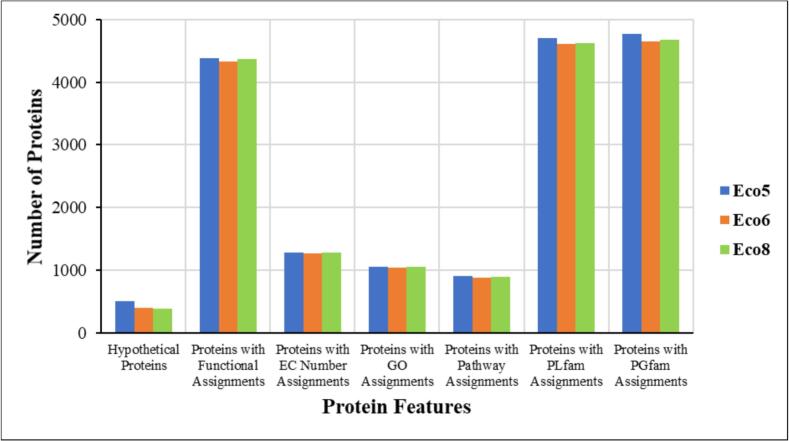
Distribution of protein features in three *E. coli* isolates**.**

Analysis of protein features across the three *E. coli* isolates revealed a high degree of functional conservation, particularly among pathway-related proteins and enzymes (Fig. 2 and Table 4).

**Table 4:** Functional genomics of the three *E. coli* isolates.

Protein features	Eco5	Eco6	Eco8
Hypothetical proteins	506	398	389
Proteins with functional assignments	4387	4335	4367
Proteins with EC number assignments	1283	1263	1281
Proteins with GO assignments	1054	1036	1053
Proteins with Pathway assignments	899	881	898
Proteins with PATRIC genus-specific family (PLfam) assignments	4698	4614	4621
Proteins with PATRIC cross-genus family (PGfam) assignments	4770	4653	4681

## 4 Distribution of the genome annotations

**Fig. 3:**
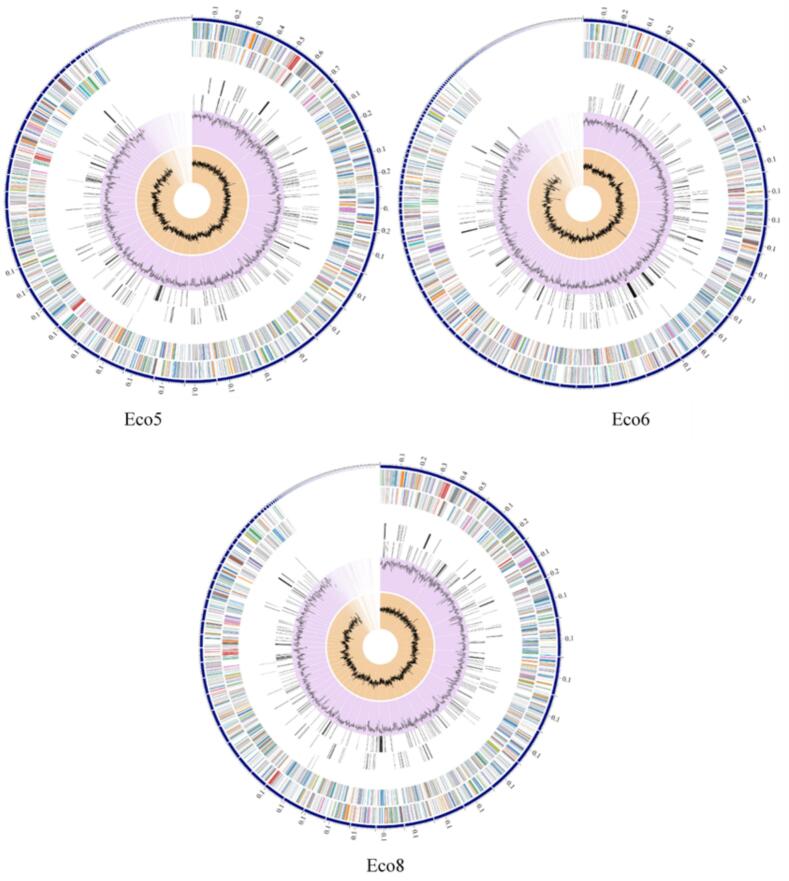
The dispersal of the genome annotations of the three *E. coli* isolates is represented on a circular graphical display. The following rings can be read out (outer to inner): contigs, forward-strand CDSs, reverse-strand CDSs, RNA genes, CDSs with homology to known antimicrobial resistance genes, CDSs with homology to known virulence factors, GC content, and GC skew.

The genome annotations and the distribution of the three *E. coli* isolates are represented in a circular graphical display (Fig. 3).

### 4.1 Subsystem analysis

**Fig. 4:**
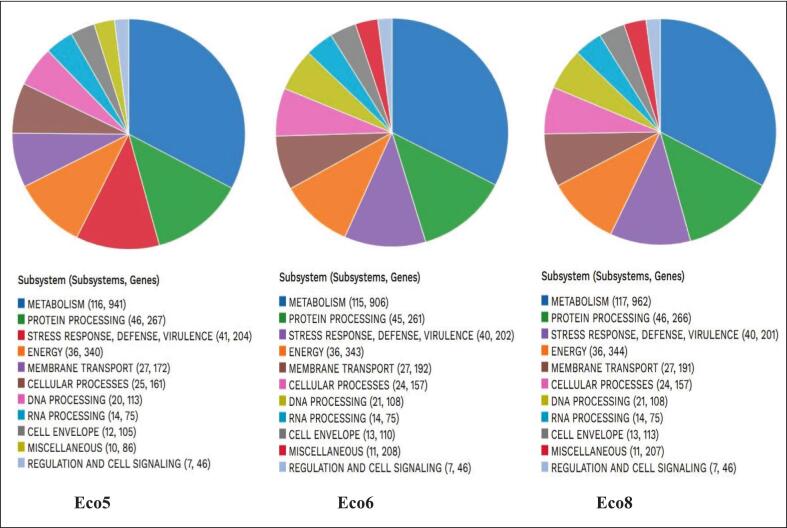
Subsystem outlines of three *E. coli* genomes.

Cellular metabolism comprises essential biological pathways, including glycolysis, the TCA cycle, and biosynthetic pathways (Fig. 4). Protein processing encompasses protein folding mechanisms and proteases. Antimicrobial and virulence mechanisms, as well as stress responses, enable microorganisms to withstand adverse environmental conditions and evade host defense mechanisms. Oxidative phosphorylation and ATP synthesis are used for energy generation. Membrane transport encompasses the movement of nutrients and ions, as well as the removal of waste. DNA replication, repair, and recombination are involved in DNA processing. Cellular processes involve structural maintenance, signaling, and cell division. Transcription, RNA modification, and the lysis system are components of RNA processing. The cell envelope includes genes involved in membrane and cell wall biosynthesis. Miscellaneous captures diverse subsystems that do not fit into other categories. Regulation and cell signaling are related to regulatory networks and signal transduction pathways.

### 4.2 Specialty genes

The dataset in Table 5 shows the variation in specialty genes, with Eco6 having the highest count (7) and Eco5 and Eco8 having lower counts. Antibiotic resistance remained quite stable across the three datasets, with the CARD and PATRIC sources showing consistent values (76–77 for CARD and 61–62 for PATRIC). Drug targets are relatively similar across the three datasets for both the Drug Bank (385–394) and the TTD (59). Transporters for the three *E. coli* isolates: TCDB has slightly fewer genes in Eco6 (871) than in Eco5 (885) and Eco8 (873). With respect to virulence factors, compared with those for Eco5 and Eco8, PATRIC-VF and VFDB tended to have fewer genes for Eco6, but the number for Victors remained similar across all three datasets.

**Table 5:** Specialty genes of three *E. coli* isolates.

Specialty genes		Eco5	Eco6	Eco8
	Source	Gene		
	Victors	4	7	2
Antibiotics Resistance	CARD	77	76	76
Antibiotics Resistance	PATRIC	62	61	61
Drug target	DrugBank	394	385	386
Drug target	TTD	59	59	59
Transporter	TCDB	885	871	873
Virulence Factors	PATRIC-VF	225	205	207
Virulence Factors	VFDB	90	68	68
Virulence Factors	Victors	243	219	222

### 4.3 Antimicrobial resistance genes

Antimicrobial resistance (AMR) mechanisms and their associated genes are outlined in Table 6. These mechanisms mark various approaches employed by bacteria to avoid antibiotic effects, ranging from enzymatic modification to efflux pumps and regulatory pathways.

**Table 6:** Antimicrobial resistance mechanisms and their associated genes.

AMR mechanism	Genes
Antibiotic activation enzyme	**KatG:** Activates pro-antibiotics, such as isoniazid, by catalyzing oxidative reactions.
Antibiotic inactivation enzyme	**BlaEC family:** Encodes β-lactamases, which make β-lactam antibiotics ineffective.
Genes of antibiotic resistance (cluster, operon, cassette)	**MarR, MarB, and MarA:** Regulate multidrug resistance through the Mar (multiple antibiotic resistance) operon.
Target of antibiotics in susceptible species	**S12p, dxrm, Alr, Ddl, folA, EF-G, gyrA, EF-Tu, Dfr, folP, gyrB, inhA, fabI, rpoB, kasA, Iso-tRNA, rho, rpoC, S10p, and MurA:** Encode essential bacterial proteins targeted by antibiotics.
Protein protection of the antibiotic target	**BcrC:** Protects antibiotic targets, often by modifying their structure to prevent antibiotic binding.
Antibiotic resistance using the efflux pump	**AcrEF-TolC, EmrAB-TolC, AcrAD-TolC, AcrZ, AcrAB-TolC,, MacA, EmrD, EmrE, MdtABC-TolC, EmrKY-TolC, MacB, MdfA/Cmr, MdtEF- TolC/OpmH, TolC, MdtL, MdtM, and SugE:** Actively pump out antibiotics from bacterial cells.
Absence of a resistance gene	**gidB:** Lack of this gene confers resistance to aminoglycosides due to the inability to methylate ribosomal RNA.
Resistance conferred by proteins that alter cell wall charge	**GdpD and PgsA:** Modify the charge of the bacterial cell wall, reducing antibiotic binding or uptake.
Regulators that modulate the expression of antibiotic resistance genes	**EmrAB-TolC, H-NS, AcrAB-TolC, GadE, and OxyR:** Modulate gene expression, leading to antibiotic resistance through transcriptional regulation.

### 4.4 Phylogenetic analysis of the bacterial isolates

**Fig. 5:**
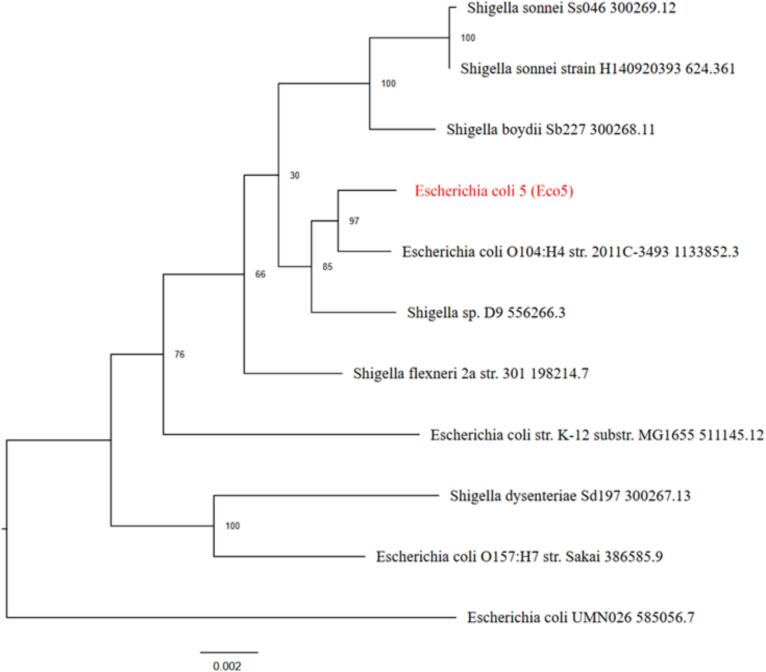
Phylogenetic analysis tree of *Escherichia coli* isolate 5 (Eco5). Mash/MinHash was used to identify representative genomes and the closest reference. From these genomes, PGFams were chosen to identify the phylogenetic placement of the target genome. MUSCLE was used to align protein sequences. The nucleotide and amino acid alignments were concatenated into a data matrix and analyzed using RAxML. Fast bootstrapping was used to generate support values for the resulting phylogenetic tree.

**Fig. 6:**
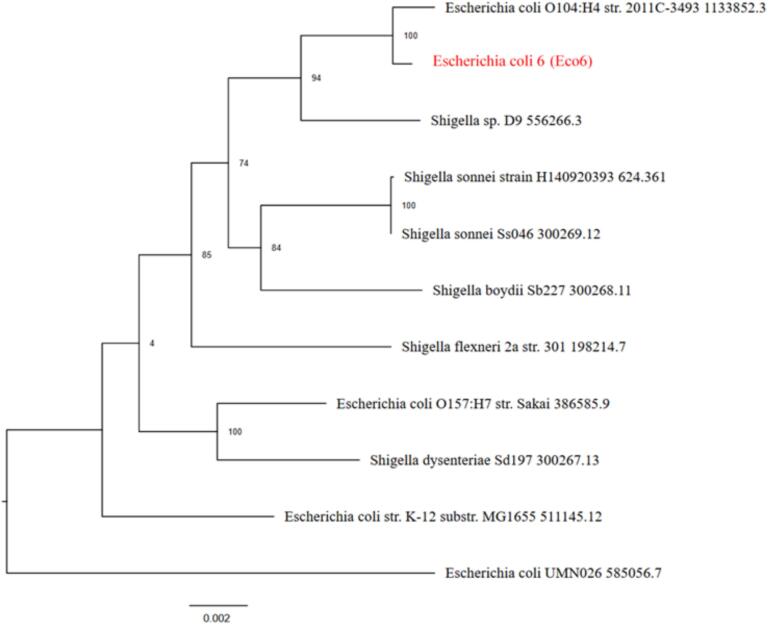
Phylogenetic analysis tree of *Escherichia coli* isolate 6 (Eco6). Mash/MinHash was used to identify representative genomes and the closest reference. From these genomes, PGFams were chosen to identify the phylogenetic placement of the target genome. MUSCLE was used to align protein sequences. The nucleotide and amino acid alignments were concatenated into a data matrix and analyzed using RAxML. Fast bootstrapping was used to generate support values for the resulting phylogenetic tree.

**Fig. 7:**
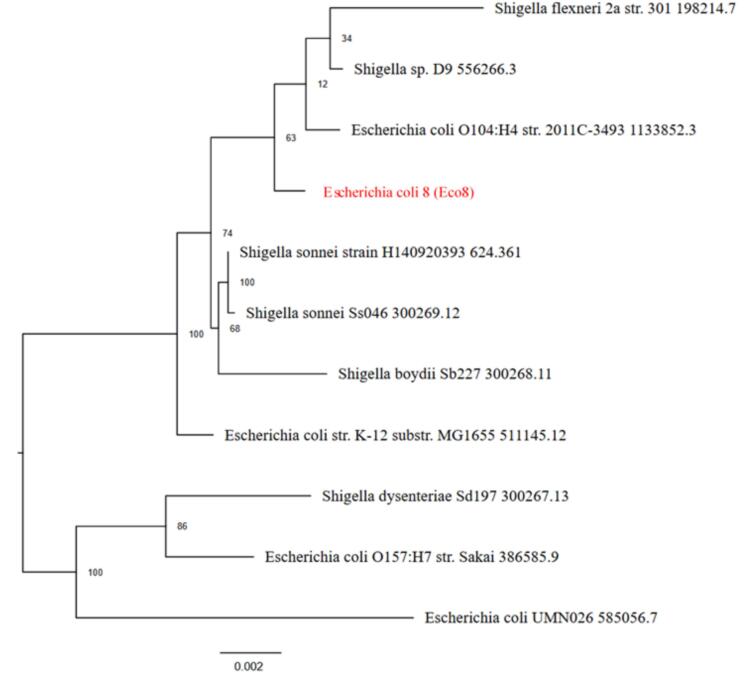
Phylogenetic analysis tree of *Escherichia coli* isolate 8 (Eco8). Mash/MinHash was used to identify representative genomes and the closest reference. From these genomes, PGFams were chosen to identify the phylogenetic placement of the target genome. MUSCLE was used to align protein sequences. The nucleotide and amino acid alignments were concatenated into a data matrix and analyzed using RAxML. Fast bootstrapping was used to generate support values for the resulting phylogenetic tree.

The results of the phylogenetic analysis revealed that Eco5 is clustered within the *E. coli* group and shares a common ancestor with E. coli O104:H4 str. 2011C-3493. The bootstrap values supporting its placement in the cluster are relatively high (97), as shown in Fig. 5. On the other hand, Eco6 appeared closely related to E. coli O104:H4 str. 2011C-3493, with strong bootstrap support (100). Its position differs slightly from that of phylogenetic Tree 1 (Fig. 6). Eco8 is positioned near the E. coli O104:H4 strain. 2011C-3493 with moderate bootstrap support (63). Compared with Eco5 and Eco6, this sample forms a distinct branch (Fig. 7). All three samples were more closely related to the E. coli group than to the Shigella clade.

## 5 Discussion

Public health requires the rapid and accurate assessment of microorganisms in drinking water and in raw and processed foods to mitigate the risks of microbial contamination and related epidemics. The detection of waterborne and foodborne pathogens is challenging because of their low concentrations. Although coliforms, including E. coli, rarely cause illness directly, their prevalence in human and warm-blooded animal feces makes them reliable microbial indicators for the potential presence of enteropathogenic bacteria in water and food supplies.^32^ Therefore, universal 16S rRNA^33^primers (Heuer et al., 1997) and mitochondrial cytochrome *c* oxidase subunit I (COI) gene primers^34^ were used, followed by sequencing, to identify the microorganisms accurately. Species-specific primers such as the *uidA* gene were also used for the rapid identification of *E. coli strains* isolated from vegetables*.* The *uidA* gene in *E. coli* encodes the β-D-glucuronidase enzyme*.* It is typically used in diagnostic assays as a specific marker for identifying *E. coli*.^35^

To our knowledge, this study is the first to report whole-genome sequencing and analysis of E. coli isolates recovered from vegetables in Iraq. Genomic data from foodborne pathogens in our region, where molecular surveillance is limited, are essential for creating a baseline for epidemiological investigation. Despite only three *E. coli* isolates being recovered from 50 samples, all three underwent WGS, confirming complete characterization of the detected *E. coli* isolates. The low recovery rate (6%) of *E. coli* isolates from the tested samples reflects the true prevalence, and such results are important for understanding the microbiological safety of fresh vegetables.

The absence of virulence genes such as *sxt1*, *sxt2*, and *eae* in the examined *E. coli* isolates may be due to the presence of a commensal, nonpathogenic isolate. These findings are consistent with those of previous reports showing a low prevalence of key virulence genes in *E. coli* isolates recovered from vegetables, highlighting the lower pathogenic potential of these strains.^36^, ^37^

One of the important molecular tools for typing microorganisms is multilocus sequence typing (MLST). In the current study, the sequence of the Eco5 isolate was ST223, which is associated with pathogenic *E. coli* isolates that cause extraintestinal infections, including urinary tract and bloodstream infections.^38^ The Eco6 and Eco8 isolates were allocated to ST1737, a less frequently reported lineage,^39^ indicating possible outbreaks or shared sources.^40^ Furthermore, the genetic diversity between ST223 and ST1737 underscores the genetic flexibility of *E. coli* and its ability to adapt to diverse environmental niches.

Genome assembly, annotation, functional analysis, and phylogenetic tree construction were performed by the Bacterial and Viral Bioinformatics Resource Center (BV-BRC). BV-BRC combines the data, technology, and extensive user communities from three BRC resources: the PAThosystems Resource Integration Center PATRIC,^41^ the Influenza Research Database (IRD),^42^ and the Virus Pathogen Database and Analysis Resource (ViPR).^43^ These platforms provide broad capabilities for genomic annotation and contain extensive datasets on protein structure, function, antimicrobial resistance, and epidemiological information.

The results of the genome assembly of the three isolates revealed that the GC content was approximately 50.6% and that the genome size ranged from approximately 4.5–5.0 Mb, which are consistent with those of known reference strains. These genomic features confirm the quality and completeness of the sequence assemblies and the accuracy of the comparative analysis. Differences in gene annotation may reflect isolate-specific adaptations or ecological niches.^44^

Functional analysis of differences in coding sequences (CDs) may reveal unique genes that contribute to isolation-specific traits such as metabolism, virulence, and adaptation to different environmental conditions.^45^ Furthermore, repeat regions may harbor regulatory elements or mobile genetic elements, influencing genomic evolution.^46^ Variation in rRNA genes may impact ribosomal efficiency and adaptation to different growth conditions.^47^

Functional genome annotation revealed extensive representation of genes involved in transport systems, stress responses, metabolic processes, and cellular regulation. The large number of proteins with enzymatic functions and their roles in metabolic pathways reflect the metabolic versatility of *E. coli*, enabling survival under miscellaneous environmental conditions.^48^ The presence of many transporter proteins further supports the adaptive capacity of these organisms, particularly in changing environments such as fresh produce surfaces.^49^ Such physiological flexibility contributes to the persistence of bacteria in food production and distribution systems and may increase the likelihood of contamination events.^50^ The number of drugs in the DrugBank is fairly high across the ecosystem, underscoring the prevalence of a wide range of potential drugs.^51^

Virulence determinants are essential for understanding a pathogen's pathogenicity. The noticeable decline from Eco5 (225) to Eco6 (205) and Eco8 (207) indicates isolate-specific differences in virulence-related genes. A low PATRI-VF count indicates reduced virulence determinants. These variations may be due to specific adaptations or variations in the mechanism of pathogenesis.^52^ Differences in virulence profiles may influence their ability to cause disease; however, the presence of multiple virulence factors indicates the potential for opportunistic pathogenesis (Liu et al., 2023).

Antimicrobial resistance (AMR) remains a critical global health concern and is driven by the extensive use and misuse of antibiotics in clinical, agricultural, and environmental settings. In the present study, the presence of AMR-related genes indicates a possible role for environmental and foodborne bacteria as reservoirs of resistance determinants. β-lactamases, such as those in the BlaEC family, hydrolyze β-lactam antibiotics. In contrast, multidrug efflux pumps, such as AcrAB-TolC, contribute to antibiotic resistance by actively exporting them from bacterial cells.^53^ Regulatory systems such as the Mar operon further increase bacterial survival under antibiotic pressure by modulating gene expression and membrane permeability.^54^ Despite the identification of multiple AMR-associated genes, including efflux pumps and regulatory elements, all E. coli isolates were phenotypically susceptible to the antimicrobial agents tested. This apparent difference between genotypic detection and phenotypic susceptibility has been reported in recent antimicrobial resistance studies and reflects the complex regulation of resistance expression. Several genes detected in bacterial genomes represent essential structural or regulatory components that do not confer resistance unless specific mutations, overexpression, or environmental triggers occur,^55^, ^56^ Genotype‒phenotype differences have been observed in gram-negative bacteria, in which resistance determinants are present despite their susceptibility to certain antibiotics.^57^ Therefore, the detection of AMR-related genes in *E. coli* isolates may reflect latent resistance potential rather than active resistance, indicating a currently low level of antimicrobial resistance expression among vegetable-associated *E. coli* isolates and underscoring the need for continued surveillance.

Phylogenetic studies of Escherichia coli and closely related species revealed overlapping genomic features because of their evolutionary proximity.^38^ The close relationship between Samples 5, 6, and 8 and *E. coli* O104:H4 aligns with reports that pathogenic *E. coli* strains share genetic similarity with Shigella.^39^

## 6 Conclusions

The results of the current study provide novel data from this region on *E. coli* isolates recovered from fresh vegetables, a potential source of foodborne pathogen transmission. Despite the small number of *E. coli* isolates (only 3) subjected to WGS, this work provides a baseline for the genomic integration of foodborne *E. coli* in Iraq. Analysis of MLST among three *E. coli* isolates revealed genetic diversity, highlighting the continuous need for genomic surveillance to monitor emerging lineages and their impact on public health. Recent advancements in bioinformatics, high-throughput sequencing, and computational tools have been critical in expanding these annotations. Variations in metabolic genes can indicate differences in metabolic capabilities and environmental adaptations. Variations in stress responses, defense, and virulence may reflect differences in pathogenic potential or in stress-adaptation strategies. Differences in energy-related genes can affect an organism's energy generation efficiency. Metabolic mutations can help bacteria resist drug treatment by altering energy-production pathways. The presence of membrane transport genes indicates an organism’s capacity for resource acquisition and waste disposal, impacting overall fitness. There were variations in cellular processes (replication and signaling pathways) among the three *E. coli* isolates. DNA processing affects genomic integrity and adaptability, both of which are critical under stress or mutagenic conditions. Genetic loci that influence transcription factors and impact DNA processing and gene regulation have been identified. Changes in RNA-processing genes can influence protein expression dynamics. The cell envelope is key to maintaining cellular integrity and responding to environmental changes. Regulation and cell signaling enable adaptability by modulating gene expression in response to stimuli. Quantification of mRNA and protein levels in single cells provides insights into the mechanisms of gene regulation.

Bacterial resistance mechanisms to antimicrobials, including efflux pumps, enzymatic modification, and regulatory systems, highlight the adaptability of bacterial genomes to withstand antimicrobial exposure stress.

## 7 Funds

None.

## Author Contribution

Z. A. T., Y. I. M., and M. R. J. designed the study and performed the work. I. A. A. and Z. A. A. analyzed the data. A. A. and S. A.S. A. wrote the manuscript draft. S.M.H.A. prepared the figures and tables. All authors review the manuscript text.

## CRediT authorship contribution statement

**Zaid Akram Thabit:** . **Yaseen Ismael Mamoori:** . **May Ridha Jaffer:** . **Ibarahim Abdulla Ahmed:** . **Zahraa Abdulkareem AlShaheeb:** . **Athraa Abdulhadi:** Writing – original draft, Visualization, Funding acquisition. **Safaa A.S. Al-Qaysi:** . **Sana M.H. AL-Shimmary:** Writing – review & editing, Software.

## Declaration of competing interest

The authors declare that they have no known competing financial interests or personal relationships that could have appeared to influence the work reported in this paper.
